# Strong- and Weak-Universal Critical Behaviour of a Mixed-Spin Ising Model with Triplet Interactions on the Union Jack (Centered Square) Lattice

**DOI:** 10.3390/e20020091

**Published:** 2018-01-29

**Authors:** Jozef Strečka

**Affiliations:** Department of Theoretical Physics and Astrophysics, Institute of Physics, Faculty of Science, P. J. Šafárik University, Park Angelinum 9, 040 01 Košice, Slovak Republic; jozef.strecka@upjs.sk

**Keywords:** mixed-spin ising model, triplet interaction, weak-universal critical behaviour, 05.50.+q, 75.10.Hk, 75.40.Cx

## Abstract

The mixed spin-1/2 and spin-*S* Ising model on the Union Jack (centered square) lattice with four different three-spin (triplet) interactions and the uniaxial single-ion anisotropy is exactly solved by establishing a rigorous mapping equivalence with the corresponding zero-field (symmetric) eight-vertex model on a dual square lattice. A rigorous proof of the aforementioned exact mapping equivalence is provided by two independent approaches exploiting either a graph-theoretical or spin representation of the zero-field eight-vertex model. An influence of the interaction anisotropy as well as the uniaxial single-ion anisotropy on phase transitions and critical phenomena is examined in particular. It is shown that the considered model exhibits a strong-universal critical behaviour with constant critical exponents when considering the isotropic model with four equal triplet interactions or the anisotropic model with one triplet interaction differing from the other three. The anisotropic models with two different triplet interactions, which are pairwise equal to each other, contrarily exhibit a weak-universal critical behaviour with critical exponents continuously varying with a relative strength of the triplet interactions as well as the uniaxial single-ion anisotropy. It is evidenced that the variations of critical exponents of the mixed-spin Ising models with the integer-valued spins *S* differ basically from their counterparts with the half-odd-integer spins *S*.

## 1. Introduction

One of the most important concepts elaborated in the theory of phase transitions and critical phenomena is universality hypothesis, which states that a critical behaviour does not depend on specific details of a model but only upon its spatial dimensionality, symmetry and number of components of the relevant order parameter. The foremost consequence of the universality hypothesis is that the critical behaviour of very different models may be characterized by the same set of critical exponents and one says that the models with the identical set of critical exponents belong to the same universality class. However, there exists a few exactly solved models whose critical exponents do depend on the interaction parameters and thus contradict the usual universality hypothesis [1]. The spin-1/2 Ising model with a three-spin (triplet) interaction on planar lattices belongs to paradigmatic exactly solved models of this type. As a matter of fact, the exact solutions for the spin-1/2 Ising model with the triplet interaction gave rigorous proof for different sets of critical exponents on different planar lattices [2,3,4,5,6,7]. More specifically, the critical exponent α for the specific heat fundamentally differs when this model is defined on centered square lattice (α=1/2) [2], triangular lattice (α=2/3) [3,4,5], decorated triangular [6], honeycomb and diced lattices [7] (α≈0, logarithmic singularity). In addition, the spin-1/2 Ising model with the triplet interaction on a kagomé lattice [8] does not display a phase transition at all.

In the present work, we will consider and exactly solve the mixed spin-1/2 and spin-*S* Ising model with the triplet interaction on the Union Jack (centered square) lattice by establishing a rigorous mapping correspondence with the symmetric (zero-field) eight-vertex model. The investigated model generalizes the model originally proposed and examined by Urumov [9] when accounting for the additional uniaxial single-ion anisotropy acting on the spin-*S* atoms. It will be demonstrated hereafter that the critical exponents of the mixed spin-1/2 and spin-*S* Ising model with the triplet interaction on the centered square lattice fundamentally depend on the interaction anisotropy, the uniaxial single-ion anisotropy, as well as, the spin parity.

## 2. Model and Exact Solution

**Figure 1:**
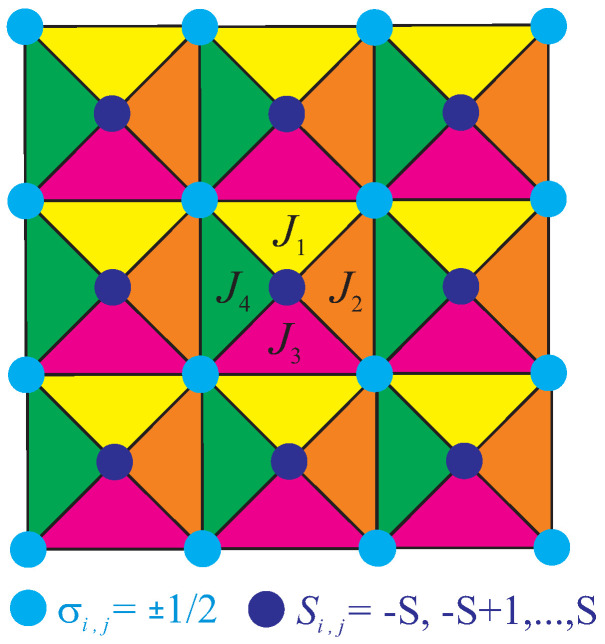
A schematic illustration of the mixed spin-1/2 (light blue) and spin-*S* (dark blue) Ising model on a centered square lattice. Four different colors are used to distinguish triplet interactions $J_{1}$, $J_{2}$, $J_{3}$ and $J_{4}$ within down-, left-, up- and right-pointing triangles, respectively.

Let us introduce the mixed spin-1/2 and spin-*S* Ising model with pure three-spin (triplet) interactions on a centered square lattice defined through the Hamiltonian:

(1) $$\begin{array}{c} H=-J_{1}\;\sum_{i,j}^{▿}\;S_{i,j}\sigma_{i,j}\sigma_{i+1,j}-J_{2}\;\sum_{i,j}^{⊲}\;S_{i,j}\sigma_{i+1,j}\sigma_{i+1,j+1}-J_{3}\;\sum_{i,j}^{△}\;S_{i,j}\sigma_{i,j+1}\sigma_{i+1,j+1}-J_{4}\;\sum_{i,j}^{⊳}\;S_{i,j}\sigma_{i,j}\sigma_{i,j+1}-D\;\sum_{i,j}\;S_{i,j}^{2}, \end{array}$$

whereas the spin-1/2 atoms (light blue circles in Figure 1) represented by the Ising spin variables $\sigma_{i,j}=\pm 1/2$ are placed at corners of a square lattice, the spin-*S* atoms (dark blue circles in Figure 1) are situated in the middle of square plaquettes. The Hamiltonian (1) takes into account four different triplet interactions $J_{1}$, $J_{2}$, $J_{3}$ and $J_{4}$ within down-, left-, up- and right-pointing triangles, respectively, in addition to the uniaxial single-ion anisotropy *D* acting on the spin-*S* atoms.

The Hamiltonian (1) can be alternatively rewritten as a sum of cell Hamiltonians $H=\sum_{i,j}^{□}H_{i,j}$, whereas the cell Hamiltonian $H_{i,j}$ involves all interactions terms depending on the central spin $S_{i,j}$:

(2) $$\begin{array}{c} H_{i,j}=-J_{1}\;S_{i,j}\sigma_{i,j}\sigma_{i+1,j}-J_{2}\;S_{i,j}\sigma_{i+1,j}\sigma_{i+1,j+1}-J_{3}\;S_{i,j}\sigma_{i,j+1}\sigma_{i+1,j+1}-J_{4}\;S_{i,j}\sigma_{i,j}\sigma_{i,j+1}-DS_{i,j}^{2}. \end{array}$$

The partition function of the mixed spin-1/2 and spin-*S* Ising model with triplet interactions on a centered square lattice can be then cast into the following form:

(3) $$\begin{array}{c} Z=\sum_{\left\{\sigma_{i,j}\right\}}\prod_{i,j}\sum_{S_{i,j}=-S}^{S}\exp\left(-\beta H_{i,j}\right)=\sum_{\left\{\sigma_{i,j}\right\}}\prod_{i,j}\omega \left(\sigma_{i,j},\sigma_{i+1,j},\sigma_{i+1,j+1},\sigma_{i,j+1}\right), \end{array}$$

where the summation $\sum_{\left\{\sigma_{i,j}\right\}}$ runs over all available spin configurations of the spin-1/2 atoms, $\beta \;=\;1/(k_{B}T)$, $k_{B}$ is Boltzmann’s constant, *T* is the absolute temperature and the expression ω denotes the Boltzmann’s weight obtained after tracing out degrees of freedom of the central spin-*S* atom:

(4) $$\begin{array}{c} \omega \left(a,b,c,d\right)=\sum_{n=-S}^{S}\exp\left(\beta Dn^{2}\right)\cosh\left[\beta n\left(J_{1}\;ab+J_{2}\;bc+J_{3}\;cd+J_{4}\;da\;\right)\right]. \end{array}$$

**Figure 2:**
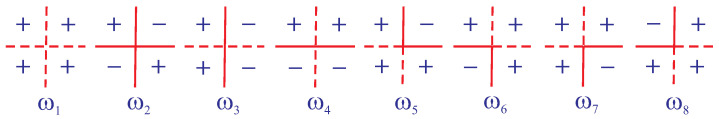
A schematic representation of two-to-one mapping correspondence between the Ising spin configurations and line coverings of the equivalent eight-vertex model on a dual square lattice (the sign ± marks σ=±1/2).

An invariance of the Boltzmann’s factor ω(a,b,c,d)=ω(−a,−b,−c,−d) implies that there exist at most eight different Boltzmann’s weights obtained from Equation (4) by considering all 16 spin configurations of the four corner spins involved therein. Hence, it follows that one may establish two-to-one mapping correspondence between a spin configuration and a relevant graph representation of the equivalent eight-vertex model on a dual square lattice according to the scheme shown in Figure 2. A solid line is drawn on a respective edge of a dual square lattice lying in between two unequally aligned neighbouring spins, while a broken line is drawn otherwise. It turns out, moreover, that the effective Boltzmann’s weights obtained after inserting all possible spin configurations of the four corner spins into Equation (4) are pairwise equal to each other:

(5) $$\begin{array}{c} \omega_{1}\left(+,+,+,+\right)=\omega_{2}\left(+,-,+,-\right)=\sum_{n=-S}^{S}\exp\left(\beta Dn^{2}\right)\cosh\left[\frac{\beta n}{4}\left(J_{1}+J_{2}+J_{3}+J_{4}\right)\right],\\ \omega_{3}\left(+,-,-,+\right)=\omega_{4}\left(+,+,-,-\right)=\sum_{n=-S}^{S}\exp\left(\beta Dn^{2}\right)\cosh\left[\frac{\beta n}{4}\left(J_{1}-J_{2}+J_{3}-J_{4}\right)\right],\\ \omega_{5}\left(-,+,+,+\right)=\omega_{6}\left(+,+,-,+\right)=\sum_{n=-S}^{S}\exp\left(\beta Dn^{2}\right)\cosh\left[\frac{\beta n}{4}\left(J_{1}-J_{2}-J_{3}+J_{4}\right)\right],\\ \omega_{7}\left(+,+,+,-\right)=\omega_{8}\left(+,-,+,+\right)=\sum_{n=-S}^{S}\exp\left(\beta Dn^{2}\right)\cosh\left[\frac{\beta n}{4}\left(J_{1}+J_{2}-J_{3}-J_{4}\right)\right], \end{array}$$

which means that the mixed-spin Ising model with triplet interactions on a centered square lattice is equivalent with the symmetric (zero-field) eight-vertex model exactly solved by Baxter [10,11]. Owing to this fact, one may easily prove an exact mapping relationship between the partition functions of the mixed-spin Ising model with triplet interactions on a centered square lattice and the zero-field eight-vertex model on a dual square lattice:

(6) $$\begin{array}{c} Z\left(\beta ,J_{1},J_{2},J_{3},J_{4},D\right)=2Z_{8-\mathrm{vertex}}\left(\omega_{1},\omega_{3},\omega_{5},\omega_{7}\right). \end{array}$$

It is apparent from the mapping relation in Equation (6) between the partition functions that the mixed-spin Ising model with triplet interactions on a centered square lattice becomes critical only if the corresponding zero-field eight-vertex model becomes critical as well. Bearing this in mind, the critical points of the mixed-spin Ising model with triplet interactions on a centered square lattice can be readily obtained from Baxter’s critical condition [10,11] when the explicit form of the effective Boltzmann’s weights in Equation (5) is taken into consideration:

(7) $$\begin{array}{c} \omega_{1}+\omega_{3}+\omega_{5}+\omega_{7}=2\max\left\{\omega_{1},\omega_{3},\omega_{5},\omega_{7}\right\}. \end{array}$$

It should be stressed that the critical exponents for the specific heat, magnetization, susceptibility and correlation length satisfy Suzuki’s weak-universal hypothesis [12] and can be calculated from:

(8) $$\begin{array}{c} \alpha =\alpha^{\prime }=2-\pi /\mu ,\;\;\;\;\beta =\pi /16\mu ,\;\;\;\;\gamma =\gamma^{\prime }=7\pi /8\mu ,\;\;\;\;\nu =\nu^{\prime }=\pi /2\mu , \end{array}$$

where $\tan\left(\mu /2\right)={\left(\omega_{1}\omega_{3}/\omega_{5}\omega_{7}\right)}^{1/2}$ on assumption that $\omega_{1}=\max\left\{\omega_{1},\omega_{3},\omega_{5},\omega_{7}\right\}$.

**Figure 3:**
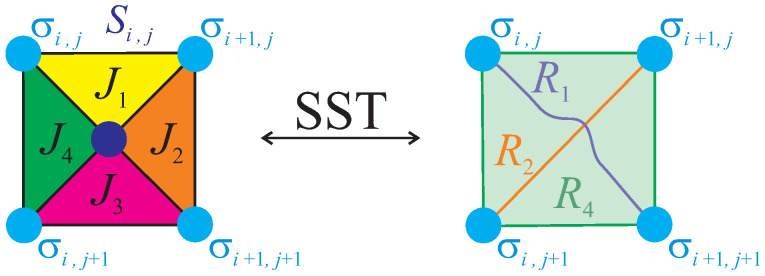
A schematic representation of the generalized star-square transformation, which replaces spin degrees of freedom of the central spin-*S* atom through two effective pair interactions ($R_{1}$, $R_{2}$) and the effective quartic interaction ($R_{4}$) between the four enclosing spin-1/2 atoms.

The exact mapping equivalence with the zero-field eight-vertex model can be alternatively proven by exploiting the spin representation of the eight-vertex model. For this purpose, the effective Boltzmann factor in Equation (4) can be replaced via the generalized star-square transformation [13,14,15] schematically drawn in Figure 3:

(9) $$\begin{array}{cc} \omega & =\sum_{n=-S}^{S}\;\;\;\exp\left(\beta Dn^{2}\right)\cosh\left[\beta n(J_{1}\sigma_{i,j}\sigma_{i+1,j}+J_{2}\sigma_{i+1,j}\sigma_{i+1,j+1}+J_{3}\sigma_{i+1,j+1}\sigma_{i,j+1}+J_{4}\sigma_{i,j+1}\sigma_{i,j})\right]\\ & =R_{0}\exp\left(\beta R_{1}\sigma_{i,j}\sigma_{i+1,j+1}+\beta R_{2}\sigma_{i+1,j}\sigma_{i,j+1}+\beta R_{4}\sigma_{i,j}\sigma_{i+1,j}\sigma_{i+1,j+1}\sigma_{i,j+1}\right). \end{array}$$

The physical meaning of the generalized star-square transformation in Equation (9) lies in replacing spin degrees of freedom related to the central spin-*S* atom through the effective pair interactions ($R_{1}$, $R_{2}$) and the effective quartic interaction ($R_{4}$) between the four enclosing spin-1/2 atoms (see Figure 3). The star-square transformation in Equation (9) must hold for any spin state of the four enclosing spin-1/2 atoms and this “self-consistency” condition unambiguously determines so far unspecified mapping parameters:

(10) $$\begin{array}{c} R_{0}={\left(\omega_{1}\omega_{3}\omega_{5}\omega_{7}\right)}^{1/4}\;,\;\;\;\beta R_{1}=\ln\left(\frac{\omega_{1}\omega_{7}}{\omega_{3}\omega_{5}}\right)\;,\;\;\;\beta R_{2}=\ln\left(\frac{\omega_{1}\omega_{5}}{\omega_{3}\omega_{7}}\right)\;,\;\;\;\beta R_{4}=4\ln\left(\frac{\omega_{1}\omega_{3}}{\omega_{5}\omega_{7}}\right). \end{array}$$

The star-square transformation in Equation (9) establishes a rigorous mapping correspondence between the partition function of the mixed-spin Ising model with triplet interactions on a centered square lattice and the partition function of the spin-1/2 Ising model on two inter-penetrating square lattices with the effective pair interactions ($R_{1}$, $R_{2}$) and the effective quartic interaction ($R_{4}$):

$$\begin{array}{c} Z\left(\beta ,J_{1},J_{2},J_{3},J_{4},D\right)=R_{0}^{2N}Z_{8-\mathrm{vertex}}\left(\beta ,R_{1},R_{2},R_{4}\right). \end{array}$$

It has been proven previously that the spin-1/2 Ising model defined on two inter-penetrating square lattices coupled together by means of the quartic interaction is nothing but the Ising representation of the zero-field eight-vertex model on a square lattice [16,17]. In this way, we have afforded alternative proof for an exact mapping equivalence between the mixed-spin Ising model with triplet interactions on a centered square lattice and the zero-field eight-vertex model on a square lattice.

## 3. Results and Discussion

**Figure 4:**
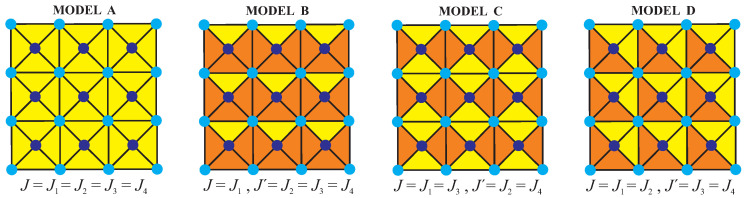
The four particular cases of the mixed spin-1/2 and spin-*S* Ising model with triplet interactions on a centered square lattice, which will be comprehensively studied in the following subsections. Different colors are used for distinguishing triplet interactions of different size.

In this section, let us discuss the most interesting results for the mixed spin-1/2 and spin-*S* Ising model with triplet interactions on a centered square lattice depending on the interaction anisotropy, the uniaxial single-ion anisotropy and the spin magnitude *S*. For the sake of simplicity, our further attention will be restricted to four particular cases to be further referred to as:

- Model A: $J\equiv J_{1}=J_{2}=J_{3}=J_{4}$,
- Model B: $J\equiv J_{1},J^{\prime }\equiv J_{2}=J_{3}=J_{4}$,
- Model C: $J\equiv J_{1}=J_{3},J^{\prime }\equiv J_{2}=J_{4}$,
- Model D: $J\equiv J_{1}=J_{2},J^{\prime }\equiv J_{3}=J_{4}$,

which will be separately treated in the following subsections. For better illustration, the four aforementioned special cases of the mixed spin-1/2 and spin-*S* Ising model with triplet interactions on a centered square lattice are schematically drawn in Figure 4, where different colors are used for distinguishing triplet interactions of different size. In addition, our subsequent discussion will be henceforth restricted to the mixed spin-1/2 and spin-*S* Ising model with both positive triplet interactions (J>0, $J^{\prime }>0$), because the other meaningful particular case with both negative triplet interactions (J<0, $J^{\prime }<0$) displays the identical critical behavior because J→−J, $J^{\prime }\to -J^{\prime }$ interchange merely causes a trivial change in a relative orientation of the nearest-neighbor spins.

### 3.1. Model A $\left(J\equiv J_{1}=J_{2}=J_{3}=J_{4}\right)$

**Figure 5:**
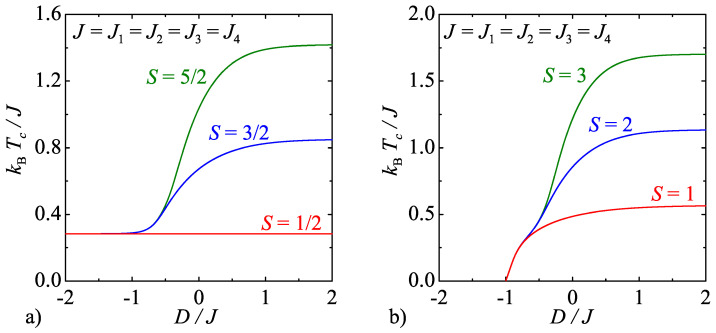
The critical temperature of the model A as a function of the uniaxial single-ion anisotropy by considering: (**a**) half-odd-integer spins *S*; and (**b**) integer spins *S*.

Let us begin with a detailed analysis of critical behaviour of the mixed spin-1/2 and spin-*S* Ising model with unique triplet interaction on a centered square lattice, which represents a very special case due to the isotropic nature of the triplet interactions $J\equiv J_{1}=J_{2}=J_{3}=J_{4}$. Under this condition, one gains from Equation (5) just two different Boltzmann’s weights:

(11) $$\begin{array}{c} \omega_{1}=\sum_{n=-S}^{S}\exp\left(\beta Dn^{2}\right)\cosh\left(\beta nJ\right),\;\omega_{3}=\omega_{5}=\omega_{7}=\sum_{n=-S}^{S}\exp\left(\beta Dn^{2}\right), \end{array}$$

where the Boltzmann’s weights in Equation (11) evidently satisfy the inequality $\omega_{1}\geq \omega_{3}$. The ordered state emergent at low enough temperature can be accordingly found from the lowest-energy states entering the Boltzmann’s weights $\omega_{1}\left(+,+,+,+\right)$ and $\omega_{2}\left(+,-,+,-\right)$. As a result, the spontaneous order has a four-fold degeneracy, because the nodal spin-1/2 atoms display either a ferromagnetic or antiferromagnetic long-range order and the central spins are oriented in such a way that either zero or two down spins appear within each triangular plaquette. This statement will remain true also for the other three particular models treated hereafter. The critical condition in Equation (7) of the mixed spin-1/2 and spin-*S* Ising model with unique triplet interaction on a centered square lattice simplifies owing to a validity of the inequality $\omega_{1}\geq \omega_{3}$ to the following form:

(12) $$\begin{array}{c} \omega_{1}=3\omega_{3}\;\Rightarrow \;\sum_{n=-S}^{S}\exp\left(\beta_{c}Dn^{2}\right)\cosh\left(\beta_{c}nJ\right)=3\sum_{n=-S}^{S}\exp\left(\beta_{c}Dn^{2}\right), \end{array}$$

where $\beta_{c}=1/\left(k_{B}T_{c}\right)$ and $T_{c}$ marks the critical temperature. It follows from Equation (8) that the critical exponents remain constant along the whole critical line in Equation (12), irrespective of the spin magnitude *S*:

(13) $$\begin{array}{c} \tan\left(\mu /2\right)=\sqrt{\omega_{1}/\omega_{3}}=\sqrt{3}\;\Rightarrow \;\alpha =\alpha^{\prime }=1/2,\;\;\;\;\beta =3/32,\;\;\;\;\gamma =\gamma^{\prime }=21/16,\;\;\;\;\nu =\nu^{\prime }=3/4, \end{array}$$

where their size is identical with the ones predicted for the spin-1/2 Ising model with the unique triplet interaction on a centered square lattice, i.e., the so-called Hintermann–Merlini model [2]. The critical temperature obtained from the numerical solution of the critical condition in Equation (12) is plotted in Figure 5 against the uniaxial single-ion anisotropy for several spin magnitudes *S*. Although the critical temperature monotonically decreases with decreasing of the uniaxial single-ion anisotropy regardless of the spin size *S*, there is fundamental difference in the critical behaviour of the mixed-spin Ising models with integer and half-odd-integer spins *S*, respectively. Namely, the critical temperature of the former mixed-spin systems becomes zero for D/J<−1 in accordance with presence of the disordered ground state, which appears due to energetic favoring of the nonmagnetic spin state S=0 of the integer-valued spins. On the other hand, the critical temperature of the latter mixed-spin systems tends towards the critical temperature of the Hintermann–Merlini model [2]$k_{B}T_{c}/J=1/4\ln\left(1+\sqrt{2}\right)\approx 0.2836\ldots$, which is achieved in the asymptotic limit D/J→−∞ (but practically already at D/J≈−1) due to energetic favoring of two lowest-valued states S=±1/2 of the half-odd-integer spins.

### 3.2. Model B $\left(J\equiv J_{1},J^{\prime }\equiv J_{2}=J_{3}=J_{4}\right)$

Next, let us relax the condition of the isotropic triplet interactions by considering the model B, where one triplet interaction (say $J\equiv J_{1}$) differs from the other three ($J^{\prime }\equiv J_{2}=J_{3}=J_{4}$). Even under this constraint, one still gets from Equation (5) just two different Boltzmann’s weights:

(14) $$\begin{array}{c} \omega_{1}=\;\;\;\sum_{n=-S}^{S}\;\;\;\exp\left(\beta Dn^{2}\right)\cosh\left[\frac{\beta n}{4}\left(J+3J^{\prime }\right)\right]\;,\;\;\;\omega_{3}=\omega_{5}=\omega_{7}=\;\;\;\sum_{n=-S}^{S}\;\;\;\exp\left(\beta Dn^{2}\right)\cosh\left[\frac{\beta n}{4}\left(J-J^{\prime }\right)\right]\;\;, \end{array}$$

which are however slightly more complicated due to the interaction anisotropy. It is evident from Equation (14) that the Boltzmann’s weights still satisfy the inequality $\omega_{1}\geq \omega_{3}$, which affords the following critical condition:

(15) $$\begin{array}{c} \omega_{1}=3\omega_{3}\;\Rightarrow \sum_{n=-S}^{S}\;\;\;\exp\left(\beta_{c}Dn^{2}\right)\cosh\left[\frac{\beta_{c}n}{4}\left(J+3J^{\prime }\right)\right]=3\;\;\;\sum_{n=-S}^{S}\;\;\;\exp\left(\beta_{c}Dn^{2}\right)\cosh\left[\frac{\beta_{c}n}{4}\left(J-J^{\prime }\right)\right]\;\;. \end{array}$$

It should be pointed out, moreover, that the critical exponents are still constants independent of the spin magnitude and the interaction anisotropy as given by Equation (13).

**Figure 6:**
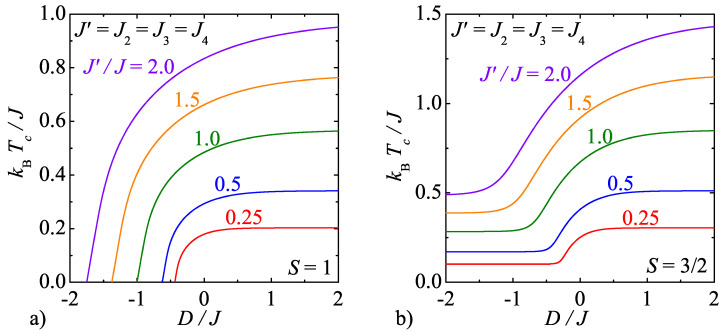
The critical temperature of the model B as a function of the uniaxial single-ion anisotropy by considering several values of the interaction anisotropy $J^{\prime }/J$ and two different spin magnitudes: (**a**) S=1; (**b**) S=3/2.

The critical frontiers of the model B are illustrated in Figure 6 for two different spin values, which demonstrate typical critical behaviour of the mixed-spin systems with half-odd-integer and integer spins *S*, respectively. It can be seen in Figure 6 that the critical temperature of the investigated mixed-spin system rises steadily with increasing of the interaction ratio $J^{\prime }/J$ both for the integer as well as half-odd-integer spins. However, it is worth remarking that the critical value of the uniaxial single-ion anisotropy needed for an onset of the disordered ground state of the mixed-spin systems with integer spins *S* shifts towards more negative values upon strengthening of the interaction ratio $J^{\prime }/J$.

### 3.3. Model C ($J\equiv J_{1}=J_{3},J^{\prime }\equiv J_{2}=J_{4}$)

Now, let us turn our attention to a critical behaviour of the model C with the triplet interactions $J\equiv J_{1}=J_{3}$, $J^{\prime }\equiv J_{2}=J_{4}$, which are pairwise equal in triangles lying in opposite to each other within elementary square cells (see Figure 4). In this particular case, one gets from Equation (5) three different Boltzmann’s weights:

(16) $$\begin{array}{cc} \omega_{1} & =\sum_{n=-S}^{S}\;\exp\left(\beta Dn^{2}\right)\cosh\left[\frac{\beta n}{2}\left(J+J^{\prime }\right)\right]\;,\;\omega_{3}=\sum_{n=-S}^{S}\;\exp\left(\beta Dn^{2}\right)\cosh\left[\frac{\beta n}{2}\left(J-J^{\prime }\right)\right]\;,\\ \omega_{5} & =\omega_{7}=\;\;\sum_{n=-S}^{S}\;\exp\left(\beta Dn^{2}\right). \end{array}$$

It directly follows from Equation (16) that the Boltzmann’s weights obey the inequality $\omega_{1}\;\geq \;\omega_{3}\;\geq \;\omega_{5}$ and, hence, the critical condition $\omega_{1}=\omega_{3}+2\omega_{5}$ can be explicitly written as follows:

(17) $$\begin{array}{c} \sum_{n=-S}^{S}\;\exp\left(\beta_{c}Dn^{2}\right)\cosh\;\left[\frac{\beta_{c}n}{2}\;\left(J+J^{\prime }\right)\right]=\;\;\sum_{n=-S}^{S}\;\exp\left(\beta_{c}Dn^{2}\right)\cosh\;\left[\frac{\beta_{c}n}{2}\;\left(J-J^{\prime }\right)\right]+2\;\;\sum_{n=-S}^{S}\;\exp\left(\beta_{c}Dn^{2}\right). \end{array}$$

Besides, the Boltzmann’s weights in Equation (16) imply that the critical exponents given by Equation (8) may display striking dependence along the critical line determined by Equation (17) on the spin magnitude, the uniaxial single-ion anisotropy as well as the interaction anisotropy according to the formulas:

(18) $$\begin{array}{c} \tan\left(\frac{\mu }{2}\right)=\frac{\sqrt{\omega_{1}\omega_{3}}}{\omega_{5}}\;\Rightarrow \;\alpha =\alpha^{\prime }=2-\frac{\pi }{\mu },\;\beta =\frac{\pi }{16\mu },\;\gamma =\gamma^{\prime }=\frac{7\pi }{8\mu },\;\nu =\nu^{\prime }=\frac{\pi }{2\mu }. \end{array}$$

**Figure 7:**
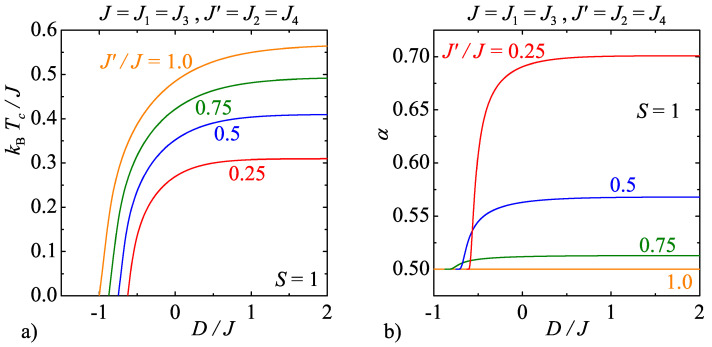
(**a**) The critical temperature of the model C as a function of the uniaxial single-ion anisotropy by considering the spin size S=1 and several values of the interaction anisotropy $J^{\prime }/J$; and (**b**) the respective changes of the critical exponent α for the specific heat along the critical lines displayed in Figure 7a.

**Figure 8:**
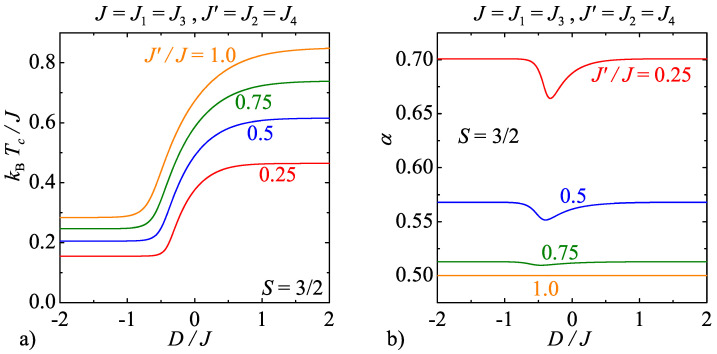
(**a**) The critical temperature of the model C as a function of the uniaxial single-ion anisotropy by considering the spin size S=3/2 and several values of the interaction anisotropy $J^{\prime }/J$; and (**b**) the respective changes of the critical exponent α for the specific heat along the critical lines displayed in Figure 8a.

For illustrative purposes, Figure 7a and Figure 8a display phase boundaries of the model C for the specific spin values S=1 and 3/2, respectively. As one can see, the same general trends can be observed in the relevant dependencies of the critical temperature on the uniaxial single-ion anisotropy and the interaction anisotropy as previously discussed for the model B. However, the model C exhibits along the displayed critical lines continuously varying critical exponents unlike the model B with the strong-universal (constant) critical exponents. For instance, it can be found in Figure 7b that the critical exponent α for the specific heat monotonically increases with increasing of the uniaxial single-ion anisotropy for the integer-valued spins S=1, whereas the observed increase is the greater the higher the interaction anisotropy is. In addition, it is quite surprising that the critical exponents for the model C with the half-odd-integer spins S=3/2 exhibit completely different weak-universal critical behaviour. Although the critical exponent α for the specific heat is shifted towards higher values upon increasing of the interaction anisotropy too, but this time the critical exponent α displays a more peculiar nonmonotonous dependence on the uniaxial single-ion anisotropy with a round minimum whose depth basically depends on the interaction anisotropy.

### 3.4. Model D $\left(J\equiv J_{1}=J_{2},J^{\prime }\equiv J_{3}=J_{4}\right)$

Finally, we will perform a comprehensive analysis of critical behaviour of the model D with the triplet interactions $J\equiv J_{1}=J_{2}$, $J^{\prime }\equiv J_{3}=J_{4}$, which are pairwise equal to each other within adjacent triangles of elementary square plaquettes. In this particular case one gets from Equation (5) three different Boltzmann’s weights given by:

$$\begin{array}{cc} \omega_{1} & =\sum_{n=-S}^{S}\;\exp\left(\beta Dn^{2}\right)\cosh\left[\frac{\beta n}{2}\left(J+J^{\prime }\right)\right]\;,\;\omega_{3}=\omega_{5}=\sum_{n=-S}^{S}\;\exp\left(\beta Dn^{2}\right),\\ \omega_{7} & =\sum_{n=-S}^{S}\;\exp\left(\beta Dn^{2}\right)\cosh\left[\frac{\beta n}{2}\left(J-J^{\prime }\right)\right]\;. \end{array}$$

It is quite apparent that the Boltzmann’s weights in Equation (19) satisfy the inequality $\omega_{1}\geq \omega_{7}\geq \omega_{3}$, which affords from Equation (7) the critical condition $\omega_{1}=2\omega_{3}+\omega_{7}$. After substituting explicit form of the Boltzmann’s weights (19) into the relevant critical condition one obtains the completely identical critical condition as given by Equation (17) for the model C. This result would suggest that the model D has exactly the same phase boundaries as the model C. However, it should be emphasized that the weak-universal critical behaviour of the model D will be characterized by different critical exponents:

(19) $$\begin{array}{c} \tan\left(\frac{\mu }{2}\right)=\sqrt{\frac{\omega_{1}}{\omega_{7}}}\;\Rightarrow \;\alpha =\alpha^{\prime }=2-\frac{\pi }{\mu },\;\beta =\frac{\pi }{16\mu },\;\gamma =\gamma^{\prime }=\frac{7\pi }{8\mu }\;\nu =\nu^{\prime }=\frac{\pi }{2\mu }, \end{array}$$

because the definition of the parameter μ governing changes of the critical exponents is different (cf. Equations (18) and (19)).

**Figure 9:**
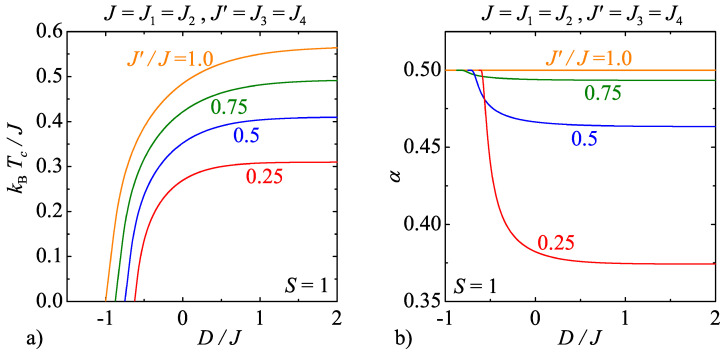
(**a**) The critical temperature of the model D as a function of the uniaxial single-ion anisotropy by considering the spin size S=1 and several values of the interaction anisotropy $J^{\prime }/J$; and (**b**) the respective changes of the critical exponent α for the specific heat along the critical lines displayed in Figure 9a.

**Figure 10:**
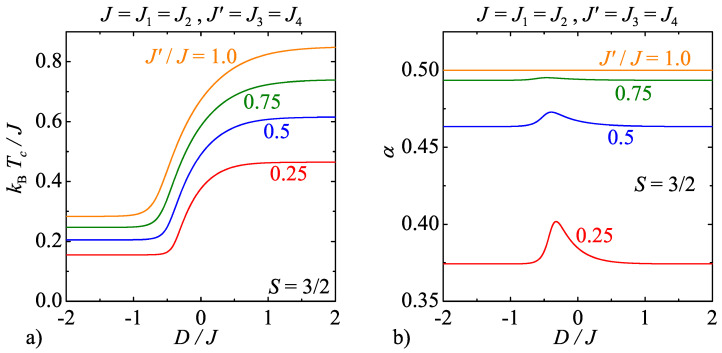
(**a**) The critical temperature of the model D as a function of the uniaxial single-ion anisotropy by considering the spin size S=3/2 and several values of the interaction anisotropy $J^{\prime }/J$; and (**b**) the respective changes of the critical exponent α for the specific heat along the critical lines displayed in Figure 10a.

For the sake of comparison, the critical temperature and critical exponent α are plotted in Figure 9 and Figure 10 for the model D by assuming two different spin values S=1 and 3/2, respectively. It has already been argued that the phase boundaries of the model D coincide with the critical lines of the model C, so let us only comment the respective behaviour of the critical exponent α for the specific heat. Figure 9b would suggest that the critical exponent α for the model D with the integer-valued spin S=1 falls down monotonically with increasing of the uniaxial single-ion anisotropy, whereas the interaction anisotropy generally reinforces the relevant decline. This behaviour is in sharp contrast with what has been previously reported for the model C, where exactly opposite tendency has been revealed (cf. Figure 7b and Figure 9b). The reduction of the critical exponent α due to the interaction anisotropy has also been detected for the model D with the half-odd-integer spin S=3/2, but this time the critical exponent α shows a peculiar nonmonotonous dependence on the uniaxial single-ion anisotropy with a round maximum. This finding is repeatedly in marked contrast with what has been previously found for the model C (c.f. Figure 8b and Figure 10b). It could be thus concluded that the models C and D display remarkably different weak-universal critical behaviour of the critical exponents even though their phase boundaries are in a perfect coincidence.

## 4. Conclusions

The mixed spin-1/2 and spin-*S* Ising model with four different triplet interactions on a centered square lattice has been exactly solved by establishing a rigorous mapping equivalence with the corresponding zero-field eight-vertex model on a dual square lattice. A rigorous proof of the aforementioned exact mapping equivalence has been afforded by two independent ways, exploiting either the graph-theoretical formulation [10,11] or the spin representation [16,17] of the zero-field eight-vertex model, the latter one obtained after adapting of the generalized star-square mapping transformation [13,14,15]. It should be mentioned that the range of applicability of the present exactly solved mixed-spin Ising model with the triplet interactions goes beyond the scope of magnetic systems, because three-body interactions might play a crucial role in determining thermodynamic behavior of other complex physical systems such fluids.

The primary goal of the present work was to examine an influence of the interaction anisotropy, the uniaxial single-ion anisotropy and the spin parity upon phase transitions and critical phenomena. It has been shown that the considered model exhibits a strong-universal critical behaviour with constant critical exponents, which are independent of the uniaxial single-ion anisotropy as well as the spin parity when considering either the isotropic model A with four equal triplet interactions or the anisotropic model B with one triplet interaction differing from the other three. On the other hand, it has also been evidenced that the models C and D with the triplet interactions, which are pairwise equal to each other, exhibit a weak-universal critical behaviour characterized by continuously varying critical exponents. Under these circumstances, the relevant critical exponents are changing along the critical lines in dependence on a relative strength of the triplet interactions as well as the uniaxial single-ion anisotropy. Although the critical boundaries of the models C and D are completely the same, different interaction anisotropy of both models is responsible for a qualitatively different weak-universal behaviour of the critical exponents. Besides, it also turns out that the mixed-spin systems with the integer-valued spins *S* exhibit very different variations of the critical exponents in comparison with their counterparts with the half-odd-integer spins *S*. A more detailed study of the order parameter and other important thermodynamic quantities, especially in a vicinity of the critical points, is left as a challenging task for our future work.
